# Not so Bad

**Published:** 1891-08

**Authors:** 


### Not So Bad.

The news has arrived that the official reports from all the Prussian clinics and pathological institutes on results of treatment with Dr. Koch's "tuberculin" have been published by the Ministry for Public Instruction. It appears that the results were much better than was believed. From the middle of November to the end of December 2,172 persons received injections, the number of injections being more than 17,500. Of these patients 932 had tuberculosis of the lungs, 120 tuberculosis of other internal organs, and 700 external tuberculosis. Of those suffering from tuberculosis of the internal organs, 13 were cured, 171 considerably improved, 194 improved, and 46 died. Of 708 patients suffering with external tuberculosis 15 were cured, 148 were considerably better, 237 better, and nine died.—*Exchange.*

